# Enteropathogens in children less than 5 years of age with acute diarrhea: a 5-year surveillance study in the Southeast Coast of China

**DOI:** 10.1186/s12879-016-1760-3

**Published:** 2016-08-20

**Authors:** Shufa Zheng, Fei Yu, Xiao Chen, Dawei Cui, Yongzhang Cheng, Guoliang Xie, Xianzhi Yang, Dongsheng Han, Yiyin Wang, Wen Zhang, Yu Chen

**Affiliations:** 1 State Key Laboratory for Diagnosis and Treatment of Infectious Diseases, Collaborative Innovation Center for Diagnosis and Treatment of Infectious Diseases, The First Affiliated Hospital, College of Medicine, Zhejiang University, No. 79, Qingchun Road, Hangzhou, 310003 People’s Republic China; 2 Key Laboratory of Clinical In Vitro Diagnostic Techniques of Zhejiang Province, No. 79, Qingchun Road, Hangzhou, 310003 People’s Republic China; 3 Center of Clinical Laboratory, Affiliated Children’s Hospital, School of Medicine, Zhejiang University, No. 57, Zhuganxiang Road, Hangzhou, 310052 People’s Republic China; 4 Department of Clinical Laboratory, Northern Jiangsu People’s Hospital, No. 98, Nantong West Road, Yangzhou, 225001 People’s Republic China

**Keywords:** Diarrhea, Enteropathogens, Children, Drug resistance

## Abstract

**Background:**

Diarrhea is the second most common cause of death among children less than 5 years of age worldwide. The etiological agents of diarrhea in the southeast coastal area of China were studied from July 2009 to December 2014.

**Methods:**

A total of the 2318 patients were enrolled in this study and examined for the presence of viruses, bacteria, and parasites. Multiplex real-time PCR was used for the detection of diarrheagenic *Escherichia.coli* (DEC). DEC strains were tested for susceptibility to a panel of 20 antibiotics using the Kirby-Bauer disc-diffusion method.

**Results:**

Of the 2318 children with diarrhea, 962 (41.5 %) were positive for at least one pathogen. Rotavirus, human calicivirus (HucV), and DEC were predominant, with detection rates of 19.1 % (443), 17.7 % (411), and 7.6 % (177), respectively. The prevalences of various pathogens in patients of different ages and in different seasons were not the same. The resistance rates of 177 strains of DEC to ampicillin, tetracycline, and cefazolin were 93.2 %, 60.0 %, and 57.7 %, respectively.

**Conclusions:**

Rotavirus, HucV, and DEC were the main pathogens associated with diarrhea in Zhejiang, China. DEC possessed high levels of antibiotic resistance. Increased monitoring of etiological agents of diarrhea is necessary.

## Background

Diarrhea is a maior cause of death in children under 5 years of age worldwide, with an estimated 1.5 million deaths per year. In developing countries, incidence rates range from 3.5 to 7.0 episodes per child per year during the first 2 years of life and from 2 to 5 episodes per child per year for the first 5 years of life [1]. Diarrhea is caused mainly by enteric pathogens, including viruses, bacteria, and parasites. There are relatively few comprehensive studies of the etiology of severe acute diarrhea in children admitted to the hospital. In China, an intensive survey of diarrheal diseases has not yet been performed, and the local characteristics of these diseases have thus been undefined. Therefore, the pathogen spectrum causing diarrhea requires investigation.

The People’s Republic of China (PRC) is located in the eastern part of Asia, facing the Pacific Ocean on the east and encompassing temperate and tropical zones. Diarrhea is one of the main life-threatening diseases in china. During the survey period, the southeast coast of China had a population of approximately 54 million and a stable annual birth rate of 500,000. An epidemiological study of an infectious disease in a community is an initial step toward the introduction of the proper interventions for controlling the disease.

## Methods

### Patients

We collected samples and clinical information from 2532 patients. A total of 214 cases were eliminated because of nonconformance with the case definition or the presence of samples that were unqualified. The 2318 patients comprised all infants and children (0 to 5 years old) admitted directly to the enteric diseases clinic of the Children’s Hospital, School of Medicine, Zhejiang University, from July 2009 to December 2014, with a diagnosis of acute diarrhea (defined as watery or loose stools ≥ 3 times per day for ≤ 14 days). The Children’s Hospital is a large tertiary pediatric center serving the Zhejiang province.

### Microbiology

Fresh fecal specimens were collected and divided into two aliquots. The first aliquot was sent to the clinical microbiology laboratory of the hospital. To identify the eggs of parasites, samples were concentrated, stained, and inoculated in different types of selective media and enrichment broths. Standard culture and identification methods were used to identify enteric pathogens. Isolated campylobacter was cultured in Columbia sheep blood AGAR inoculated with samples in a microaerophilic pot for 3–5 days at 42 °C. Five lactose-fermenting colonies suggestive of *Escherichia. coli* from each MacConkey plate were selected and stored in semisolid media. The second aliquot was tested for the presence of viruses. The detection of group A rotaviruses was carried out with an enzyme-linked immunosorbent assay (ELISA) using a commercial kit (IDEIATM Rotavirus, DAKO Ltd., UK) in accordance with the manufacturer’s recommendations. Multiplex reverse transcription-polymerase chain reaction (RT-PCR) was carried out to detect the presence of the other viruses. The reverse transcription (RT) reaction was performed using random primers according to a previous report [2]. For multiplex PCR, two sets of specific primers were used (Table 1). The first primer set included equimolar amounts of specific primers for group B rotavirus, group C rotavirus and adenovirus. The second primer set included equimolar amounts of specific primers for norovirus GI, norovirus GII, sapovirus and astrovirus. The optimized reaction system consisted of 2.5 μl of 10 × Taq Buffer, 2.0 μl of 25 mmol/L MgCl_2_, 2.0 μl of 2.5 mol/L dNTPs, 0.125 μl of 5 U/μl Taq DNA polymerase, 2.5 μl of template cDNA/DNA, 0.2 μl of 33 μmol/L virus-specific primer and ddH_2_O to bring the total reaction volume to 25 μl for each specimen. The cycling parameters were as follows: 94 °C for 5 min and 35 cycles of 94 °C for 30 s, 55 °C for 30 s, 72 °C for 1 min; and 72 °C for 7 min.

**Table 1:** Virus-specific primers used for the multiplex PCR

Virus	Primer	Polarity	Sequence(5′-3′)	Product size (bp)	Reference
Group B rotavirus	B5-2	+	GGCAATAAAATGGCTTCATTGC	814	[13]
	B5-3	-	GGGTTTTTACAGCTTCGGCT		
GroupC rotavirus	NG8S1	+	ATTATGCTCAGACTATCGCCAC	352	[13]
	NG8S2	-	GTTTCTGTACTAGCTGGTGAAC		
Adenovirus	Ad1	+	TTCCCCATGGCICAYAACAC	482	[13]
	Ad2	-	CCCTGGTAKCCRATRTTGTA		
Norovirus (GI)	G1-SKF	+	CTGCCCGAATTYGTAAATGA	330	[14]
	GI-SKR	-	CCAACCCARCCATTRTACA		
Norovirus (GII)	CoG2F	+	CARGARBCNATGTTYAGRTGGATGAG	387	[14]
	G2-SKR	-	CCRCCNGCATRHCCRTTRTACAT		
Sapovirus	SLV-5317	+	CTCGCCACCTACRAWGCBTGGTT	434	[14]
	SLV-5749	-	CGGRCYTCAAAVSTACCBCCCCA		
Astrovirus	PreCAP1	+	GGACTGCAAAGCAGCTTCGTG	719	[14]
	82b	-	GTGAGCCACCAGCCATCCCT		

### Characterization of diarrheagenic *E. coli* by multiplex PCR

Multiplex real-time PCR was used for the detection of diarrheagenic *E. coli* (DEC). The target genes, primer sequences, PCR conditions, amplification parameters, and interpretation of results were performed according to a study by Muller et al. [3]. Strains that were *escV*^+^/*bfpB*^+^ were considered as typical enteropathogenic *E. coli* (EPEC), whereas *escV*^+^/*bfpB*^-^ strains were identified as atypical EPEC. The quality control strains were EPEC CMCC44155 (*escV*: +), enterohemorrhagic (or Shiga toxin-producing) *E. coli* (STEC) EDL933 (*stx1*: +; *stx2*: +; and *escV*: +), enterotoxigenic *E. coli* (ETEC) H10407 (*elt*: +; *estIa*: +; and *estIb* +), enteroinvasive *E. coli* (EIEC) CMCC44825 (*invE*: +), and enteroaggregative *E. coli* (EAEC) O42 (*astA*: +; *aggR*: +; and *pic*: +).

### Antimicrobial susceptibility testing

DEC strains were tested for susceptibility to a panel of 20 antibiotics with the Kirby-Bauer disc-diffusion method. Results were interpreted according to the guidelines of the Clinical Laboratory Standards Institute (CLSI, 2010). The definitions of multidrug resistance were obtained from the report of Magiorakos et al. [4]. Quality control strains contained *E. coli* ATCC 25922, *Staphylococcus aureus* ATCC 25923, and *Klebsiella pneumoniae* ATCC700603 (producing extended-spectrum beta-lactamase, ESBL).

### Data analysis

Statistical analyses were performed using Statistical Package for Social Sciences (SPSS) version 17.0. The statistical significance of the differences between the groups was evaluated by the χ^2^-test or Fisher’s exact test (whenever necessary). All *p* values reported are two-sided. *p* < 0.05 was considered to be statistically significant.

## Results

From July 2009 to December 2014, a total of 2318 patients were enrolled in this study. The mean age of children with diarrhea was 1.17 ± 1.23 (SD) years. Among the patients, the ratio of males to females was 1.72:1. Basic information and clinical symptoms of patients analyzed are presented in Table 2.

**Table 2:** Basic information and clinical symptoms of the 2318 children with acute diarrhea

Characteristic	No. of case (%)
Seasons	
Spring	446(19.2)
Summer	855(36.9)
Fall	414(17.9)
Winter	603(26.0)
Age (years)	
< 1	1013(43.7)
1–2	711(30.7)
3–5	594(25.6)
Mean age ± SD	1.17 ± 1.23
Sex ratio (M/F)	1.72/1
Diarrhea	
Frequency	4.17 ± 2.43
Loose	772(33.3)
Mucus	248(10.7)
Watery	361(15.6)
Bloody	13(0.5)
Unknown	924(39.9)
Vomiting	781(32.5)
Frequency	2.28 ± 1.71
Watery	57(2.7)
Stomach contents	724(29.8)
Fever	212(8.9)
Abdominal pain	147(6.0)
Antibiotic consumption	114(4.4)

Of the 2318 children with diarrhea, 962 (41.5 %) were positive for at least one pathogen. Pathogenic viruses were isolated from 728 (31.4 %) samples, bacterial were present in 285 (12.3 %) samples, and parasites were detected in 18 (0.8 %) samples. Rotavirus, human calicivirus (HucV), and DEC were detected most often. The incidences of enteropathogens detected from the collected specimens are presented in Table 3.

**Table 3:** Incidence and age distribution of enteropathogens isolated from patients

Pathogen	No. (%) of isolates in patients	No. (%) of isolates in patients of the following ages			*p* value	No. (%) of isolates in patients of the following seasons				*p* value
		<1 year (*n* = 1013)	1–2 years (*n* = 711)	2–5 years (*n* = 594)		Spring (*n* = 446)	Summer (*n* = 855)	Fall (*n* = 414)	Winter (*n* = 603)	
Rotavirus	443(19.1)	184(18.2)	170(23.9)	89(15.0)	**0.000**	96(21.5)	81(9.5)	69(16.7)	197(32.7)	**0.000**
Calicivirus	411(17.7)	151(14.9)	123(17.3)	137(23.1)	**0.000**	101(22.6)	130(15.2)	61(14.7)	119(19.7)	**0.002**
Norovirus I	52(2.2)	14(1.4)	15(2.1)	23(3.9)	**0.005**	4(0.9)	18(2.1)	16(3.9)	14(2.3)	**0.033**
Norovirus II	320(13.8)	127(12.5)	93(13.1)	100(16.8)	**0.044**	90(20.2)	101(11.8)	41(9.9)	88(14.6)	**0.000**
Sappovirus	39(1.7)	10(1.0)	15(2.1)	14(2.4)	0.068	7(1.6)	11(1.3)	4(1.0)	17(2.8)	0.077
Astrovirus	38(1.6)	13(1.3)	12(1.7)	13(2.2)	0.383	9(2.0)	9(1.1)	5(1.2)	15(2.5)	0.145
Adenovirus	51(2.2)	22(2.2)	24(3.4)	5(0.8)	**0.008**	9(2.0)	11(1.3)	14(3.4)	17(2.8)	0.067
*Diarrheagenic E. coli*	177(7.6)	6 (6.0)	43(6.0)	73(12.3)	**0.000**	24(5.4)	98(11.5)	21(5.1)	34(5.6)	**0.000**
EAEC	104(4.5)	39(3.8)	22(3.1)	43(7.2)	**0.001**	14(3.1)	62(7.3)	7(1.7)	21(3.5)	**0.000**
EPEC	40(1.7)	8(0.8)	13(1.8)	19(3.2)	**0.002**	5(1.1)	19(2.2)	9(2.2)	7(1.2)	0.281
ETEC	24(1.0)	7(0.7)	6(0.8)	11(1.9)	0.071	5(1.1)	11(1.3)	2(0.5)	6(1.0)	0.651
EHEC	7(0.3)	5(0.5)	2(0.3)	0(0)	NA	0(0)	4(0.5)	3(0.7)	0(0)	NA
EIEC	2(0.1)	2(0.2)	0 0)	0 0)	NA	0 0)	2 0.2)	0 0)	0(0)	NA
*Shigella* spp.	34(1.5)	15(1.5)	12(1.7)	7(1.2)	0.747	4(0.9)	21(2.5)	7(1.7)	2(0.3)	**0.006**
*S. flexneri*	2(0.1)	0(0)	0(0)	2(0.3)	NA	0(0)	2(0.2)	0(0)	0(0)	NA
*S. sonnei*	26(1.1)	11(1.1)	10(1.4)	5(0.8)	0.621	4(0.9)	18(2.1)	3(0.7)	1(0.2)	**0.004**
*S. boydii*	6(0.3)	4(0.4)	2(0.3)	0(0)	NA	0(0)	1(0.1)	4(1.0)	1(0.2)	NA
*Vibrio parahaemolyticus*	29(1.3)	10(1.0)	6(0.8)	13(2.2)	0.056	2(0.4)	16(1.9)	7(1.7)	4(0.7)	0.062
*Salmonella* spp.	15(0.6)	4(0.4)	2(0.3)	9(1.5)	**0.009**	2(0.4)	9(1.1)	2(0.5)	2(0.3)	0.312
*Campylobacter jejuni*	10(0.4)	5(0.5)	2(0.3)	3(0.5)	0.764	0(0)	5(0.6)	3(0.7)	2(0.3)	NA
*Plesiomonas shigelloides*	9(0.4)	2(0.2)	2(0.3)	5(0.8)	0.115	2(0.4)	5(0.6)	2(0.5)	0(0)	NA
*Vibrio cholerae*	6(0.3)	4(0.4)	0(0)	2(0.3)	NA	0(0)	3(0.4)	0(0)	3(0.5)	NA
*Aeromonas hydrophila*	5(0.2)	2(0.2)	0(0)	3(0.5)	NA	0(0)	5(0.6)	0(0)	0(0)	NA
*Yersinia enterocolitica*	4(0.2)	5(0.5)	0(0)	0(0)	NA	1(0.2)	3(0.4)	0(0)	0(0)	NA
*Campylobacter* spp.	3(0.1)	1(0.1)	0(0)	2(0.3)	NA	0(0)	0(0)	2(0.5)	1(0.2)	NA
*Amoeba*	14(0.6)	1(0.1)	2(0.3)	10(1.7)	**0.000**	2(0.4)	8(0.9)	4(1.0)	0(0)	NA
*Giardia lamblia*	2(0.1)	0(0)	2(0.3)	0(0)	NA	0(0)	2(0.2)	0(0)	0(0)	NA
*Cryptosporidiosis*	2(0.1)	0(0)	0(0)	2(0.3)	NA	0(0)	0(0)	2(0.5)	0(0)	NA

NA, not applicable; Boldface, indicates statistical significance(p<0.05)

Of the 2318 samples examined from patients with diarrhea, rotavirus was the most common pathogen with a prevalence of 19.1 %. The detection rate in children aged 1 to 2 years was 23.9 %, significantly higher than that in children in the other groups (*p* < 0.001). Rotavirus infection was most prevalent in children during the winter (32.7 %), but Was also present in children in the spring (21.5 %) and fall (16.7 %), and the lowest infection rate was seen in the summer (9.5 %). There was a significant difference in rotavirus prevalence between seasons (*p* < 0.001).

HucV was the second most frequently identified enteric pathogen with a prevalence of 17.7 %. Of the samples positive for HucV, 320 (13.8 %) were positive for norovirusII, 52 (2.2 %) were positive for norovirusI, and 39 (1.7 %) were positive for sappovirus. HucV infection was most prevalent in children aged 2–5 years old (23.1 %). The detection rate was highest in the spring (22.6 %). There were significantly differences in infection rates in different seasons (*p* < 0.001).

The third most common pathogen in diarrheal samples was DEC, the most prevalent bacterial enteropathogen, with an isolation rate of 7.6 %. Of the samples positive for DEC, 104 (4.5 %) were positive for EAEC, 40 (1.7 %) for EPEC, 24(1.0 %) for ETEC, 7 (0.3 %) for EHEC, and 2 (0.1 %) for EIEC. The highest detection rate was found in the 2- to 5-year-old age group (12.3 %), and there were statistically significant differences between age groups (*p* < 0.001). EAEC, EPEC, and ETEC were more frequently isolated in children more than two years of age (7.2 % 3.2 % and 1.9 %, respectively), whereas EIEC and ETEC were less frequently found. The detection rate in summer (11.5 %) was higher than that in the other seasons (*p* < 0.001). No children were identified with infections of more than two DEC.

In addition, we detected adenovirus (2.2 %), astrovirus (1.6 %), *Shigella* spp. (1.5 %), *Vibrio parahaemolyticus* (1.3 %), *Salmonella* spp. (0.6 %), *Campylobacter jejuni* (0.4 %), *Plesiomonas shigelloides* (0.4 %), *Vibrio cholerae* (0.3 %), *Aeromonas hydrophila* (0.2 %), *Yersinia enterocolitica* (0.2 %), *Campylobacter* spp*.* (0.1 %), *Amoeba* (0.6 %), *Giardia lamblia* (0.1 %), and *Cryptosporidiosis* (0.1 %) in samples. The detection rates for participants of different ages and during different seasons are shown in Table 3.

The occurrences of mixed infections with enteric pathogens is shown in Table 4. Among children with diarrhea, 247 (10.7 %) were infected with two or more pathogens. The most frequent pattern of mixed infection was the combination of rotavirus with HucV (121 patients). In total, 192 specimens exhibited mixed infections associated with rotavirus, 182 specimens exhibited mixed infections associated with HucV, and 58 specimens exhibited mixed infections associated with DEC.

**Table 4:** Children with multiple pathogens isolated from a single specimen in diarrhea children

Patterns of infection	No. of case (%)
Rotavirus + Calicivirus	121(5.2)
Rotavirus + Calicivirus + *Diarrheagenic E. coli*	21(0.9)
Rotavirus + *Diarrheagenic E. coli*	19(0.8)
Calicivirus + *Diarrheagenic E. coli*	14(0.6)
Rotavirus + Adenovirus	12(0.5)
Rotavirus + Astrovirus	11(0.5)
Rotavirus + Calicivirus + Adenovirus	9(0.4)
Calicivirus + Adenovirus	7(0.3)
Calicivirus + Astrovirus	7(0.3)
Rotavirus + Calicivirus + Astrovirus	5(0.2)
Rotavirus + Vibrio parahaemolyticus	3(0.1)
Calicivirus + *Salmonella* spp.	3(0.1)
Calicivirus + *Shigella* spp.	2(0.1)
*Salmonella* spp. + *Vibrio cholerae*	2(0.1)
Calicivirus + *Vibrio parahaemolyticus*	2(0.1)
*Salmonella* spp. + Yersinia intestinal colon	1(0.0)
*Vibrio parahaemolyticus* + *Vibrio cholerae*	1(0.0)
Rotavirus + Calicivirus + Astrovirus + Adenovirus	1(0.0)
Calicivirus + Adenovirus + *Shigella* spp.	1(0.0)
*Diarrheagenic E. coli* + *Campylobacter jejuni* + *Yersinia enterocolitica*	1(0.0)
Rotavirus + Adenovirus + *Diarrheagenic E. coli*	1(0.0)
Calicivirus + *Diarrheagenic E. coli* + *Campylobacter jejuni*	1(0.0)
Rotavirus + Calicivirus + *Yersinia enterocolitica*	1(0.0)
Rotavirus + Calicivirus + Astrovirus + *Diarrheagenic E. coli*	1(0.0)

The antimicrobial resistance patterns of the DEC, expressed as a percentage of isolates are shown in Table 5. The resistance rates of 177 strains to ampicillin, tetracycline, and cefazolin were 93.2, 60.0, and 57.7 %, respectively. The resistance rates to cefepime, cefoxitin, piperacillin-tazobactam, and cefoperazone-sulbactam were all below 10 %. No resistance to imipenem or meropenem was detected. The antimicrobial resistance rates in EAEC, ETEC, and EPEC were no significantly different.

**Table 5:** Antimicrobial resistance patterns of Diarrheagenic *Escherichia coli* (DEC) isolates

Antimicrobial	DEC		EAEC		ETEC		EPEC		STEC		*P* ^a^
	(*n* = 177)		(*n* = 104)		(*n* = 40)		(*n* = 24)		(*n* = 7)		
	%R	%S	%R	%S	%R	%S	%R	%S	%R	%S	
AMP	93.2	2.8	96.2	1.9	95.0	0	83.3	4.2	71.4	28.6	0.055
TCY	60.0	38.3	61.5	38.5	55.0	37.5	79.2	20.8	0	100	0.258
CZO	57.7	26.9	55.8	27.9	55.0	27.5	79.2	20.8	28.6	28.6	0.093
SXT	57.2	39.5	63.5	33.7	55.0	37.5	50.0	50.0	0	100	0.467
PIP	51.4	32.5	49.0	31.7	55.0	27.5	62.5	33.3	28.6	71.4	0.209
CXM	46.3	50.3	51.0	49.0	37.5	55.0	45.8	54.2	28.6	28.6	0.347
SAM	45.1	37.1	42.3	36.5	55.0	37.5	45.8	29.2	28.6	71.4	0.392
CTX	42.3	54.9	49.0	51.0	27.5	62.5	41.7	54.2	28.6	71.4	0.064
GEN	35.4	60.0	36.5	59.6	37.5	55.0	37.5	58.3	0	100	0.992
ATM	21.7	68.6	27.9	63.5	10.0	90.0	20.8	54.2	0	71.4	0.070
CIP	17.7	79.4	23.1	72.1	7.5	92.5	16.7	83.3	0	100	0.095
CAZ	14.8	85.2	19.2	80.8	10.0	90.0	8.3	91.7	0	100	0.226
AMC	12.0	69.1	14.4	72.1	10.0	62.5	0.0	66.7	28.6	71.4	0.127
FEP	11.4	76.5	14.4	74.0	10.0	72.5	4.2	95.8	0	71.4	0.343
FOX	5.1	93.7	6.7	91.3	0	100	0	100	28.6	71.4	0.106
TZP	4.0	90.9	6.7	85.6	0	100	0	95.8	0	100	0.106
CSL	2.3	74.3	3.8	72.1	0	72.5	0	79.2	0	100	0.283
AMK	1.1	93.7	1.9	93.3	0	90.0	0	100	0	100	0.536
IPM	0	100	0	100	0	100	0	100	0	100	NA^b^
MEM	0	100	0	100	0	100	0	100	0	100	NA^b^

EAEC enteroaggregative *E. coli*, ETEC enterotoxigenic *E. coli*, EPEC enteropathogenic *E. coli*, STEC Shiga toxin-producing *E. coli*, R Resistance, S susceptibility, AMP Ampicillin, TCY Tetracycline, CZO Cefazolin, SXT Trimethoprim-Sulfamethoxazole, PIP Piperacillin, CXM Cefuroxime, SAM Ampicillin-Sulbactam, CTX Cefotaxime, GEN Gentamicin, ATM Aztreonam, CIP Ciprofloxacin, CAZ Ceftazidime, AMC Amoxicillin-Clavulanicacid, FEP Cefepime, FOX Cefoxitin, TZP Piperacillin-Tazobactam, CSL Cefoperazone-Sulbactam, AMK Amikacin, IPM Imipenem, MEM Meropenem. Data on Enteroinvasive *E. coli* and Coinfection are not shown because of small numbers

^a^For comparison of the resistance percentage among the different pathotypes of Diarrheagenic *E. coli.* STEC does not include, because of too few

^b^NA, not applicable

## Discussion

The State Key Laboratory for diagnosis and treatment of infectious disease at Zhejiang University is an important component of the infectious surveillance platform in China, and it is responsible for monitoring the spectrum of pathogens in Zhejiang Province and the surrounding areas. Monitoring the rates of diarrhea is an important aspect of monitoring and has always been our top priority. In the current study, at least one pathogen was isolated from 41.5 % (962/2318) of stool samples from patients with diarrhea. This percentage is lower than that in previous studies carried out in developing countries addressing the presence of virus, bacteria or parasites [5, 6]. Various factors may account for such a difference. Many studies have shown that diarrhea prevalence is strongly associated with sanitation [7–9]. Zhejiang Province located on the southeastern coast of China, is on the forefront of reform. Living standards and health conditions have greatly improved in recent years, which is the main reason for the reduction in the prevalence of diarrhea pathogens. In addition, we found that the *Proteus* detection rate in the region was up to 15 %, but the pathogenicity of *Proteus* is considered controversial [10]. Finally, antimicrobial therapy has been initiated prior to sample collection in some cases, reducing the percentage of bacterial enteropathogens isolated.

Several studies have documented the prevalence of rotavirus in children with acute gastroenteritis to be in the range of 13 to 58 % [1, 11–14]. The overall detection rate of rotavirus among children aged < 5 years was 19.1 %, which was significantly lower than rates reported in surveys conducted in many other countries in Asia, Europe, and the Americas [11, 13, 15, 16]. The main reason for this discrepancy could be sample selection bias. In the current study, the detection rate during winter was as high as 32.8 %, but the specimens collected in winter only accounted for 1/4 of all the specimens, thereby leading to a reduction in the overall detection rate. We found that the detection rate of rotavirus was higher in children under the age of 3 than in children of other ages and that it was higher in winter than in other seasons, which is consistent with other reports [15, 17, 18]. Prevention and control of rotavirus infections during winter should be emphasized, especially for infants and young children under 3 years of age. However, interestingly, other pathogens were detected in approximately 35 % of rotavirus-positive samples. Although other surveillance studies reported that rotavirus could be the putative cause of diarrhea, we would like to determine that whether rotavirus was truly the etiological agent of disease in these patients, as well as the contribution of the other pathogens to the disease process.

This survey confirms worldwide studies identifying HucV as the second most common cause of severe diarrhea in young children admitted to hospitals in developed and developing countries [19–21]. Our surveillance data indicated that HucV was responsible for 17.7 % of diarrhea cases in Zhejiang. Fang et al. reported that from 1999 to 2005, the HucV detection rate in Zhejiang region was 38.6 % [22]. Data in our study, as well as in previous studies, suggest that HucV is a common viral agent associated with childhood diarrhea in Zhejiang. The variation in detection rates is most likely related to the detection methods as well as the target subjects. In all cases of HucV infection, norovirus was present in approximately 90.4 % of cases, which was observed in Lanzhou City, China [16]. Norovirus is believed to be a major pathogen leading to diarrheal outbreak and causes symptomatic infections in older children and adults [15]. However, in the current study, we found that the detection rate of norovirus among participants younger than 3 years was lower than that among participants older than 3 years, therefore, younger children infected with norovirus should be monitored. We found that the detection rate of norovirus exhibited a seasonal trend; specifically, detection rates in the winter and spring were significantly higher than those in the summer and autumn, which is consistent with other reports [23, 24]. The current study also revealed the presence of multiple infections, as nearly half of the HucV-positive samples contained multiple infections.

DEC was the most frequently isolated bacterial enteropathogen, supporting the well-documented role of *E. coli* in diarrheal disease [6]. Our study confirmed that DEC was more frequently isolated from samples from older infants than from samples from younger infants. Quiroga et al. suggested that increased exposure to pathogens after 6 months of age were potentially resulted from the introduction of contaminated foods into the diet [25]. DEC detection rates have been reported to correlate with the seasons [26, 27]. Our study also confirmed that the detection rate of DEC in the summer is higher than in the winter. Many reports have demonstrated the association of EAEC with diarrhea in children. In this study, EAEC was demonstrated to be the most prevalent DEC subtype. The role of EPEC in diarrheal disease has been previously described in several studies, In the current study, we confirmed that EPEC is an important pathogen contributing to diarrhea in children. EPEC infection has been considered a primary disease in infants younger than 2 years of age [27], but in the current study we did not find evidence to confirm this assumption. Most epidemiological studies with data on isolation rates according to age have reported ETEC prevalence rates of 10–20 % among children <12 months of age. However, the prevalence of ETEC in Zhejiang province was 1.1 %, which is lower than previously the reported rates. The low frequencies of EHEC and EIEC strains in the studied population were, in general, in agreement with other studies performed in different parts of the world [28, 29].

Astroviruses are currently considered the third causative agent of gastroenteritis after rotavirus and norovirus [30]. In this study, astrovirus was found in 1.6 % of the stool samples, which is a slightly lower percentage than previously reported in China [30]. In the current study, adenoviruses were detected in 2.2 % of the cases, which inconsistent with the rate reported in Tunisian (2.3 %) [31], Australia (3.1 %) [32], and France (3 %) [33]. *Shigella* spp. was revealed to play an important role in the diarrhea in all age groups. The frequency of isolation of *Shigella* spp. was 1.5 % on average, which is markedly lower than the rate reported in other countries [5, 34]. Research has shown that *Shigella* spp. were more easily detected in older children [34, 35]; however, we did not find such a trend. As reported in the developed regions [36], the dominant pathogen was *S. sonnei*. Another relevant enteropathogenic bacterium, *Vibrio parahaemolyticus*, which is a food-borne enteropathogen in coastal areas [34], was found in 29 cases (1.3 %) and was ranked third among bacterial pathogens in this study. Although the incidence of *V. parahaemolyticus*-related diarrhea was not high in the present survey, attention should be paid to the treatment of seafood, which is frequently implicated as a reservoir of marine *V. parahaemolyticus.* Fifteen strains of *Salmonella* spp. were isolated from diarrheal specimens, which was lower than the number reported in other regions [14]. Additionally, in our study, the presence of *Amoeba*, one of the most important parasitic pathogens, was significantly associated with diarrhea. Detection rates of *Aeromonas hydrophila*, *Campylobacter jejuni*, *Plesiomonas shigelloides*, *Yersinia enterocolitica*, *Campylobacter* spp., *Giardia lamblia*, and *Cryptosporidiosis* were all less than 0.5 %, which is consistent with other reports [14, 37].

Mixed infections are an evolving problem in the epidemiology of diarrhea. With better diagnostic tools, more mixed infections are being identified. The presence of mixed infections complicates the identification of a specific pathogen responsible for the disease and may indicate an additive effect of each pathogen present in a co-infection. In this study, the most common mixed infection included rotavirus and calicivirus, and was present in a total of 55 (5.2 %) strains, consistent with research in Spain [13]. We also found 2 cases infected with four types of pathogens, which is rare in other reports.

Traditional antibiotics including trimethoprim-sulfamethoxazole, ampicillin, and tetracycline showed low activities against the DEC strains. The resistance to ampicillin was up to 92.4 %, higher than that in the neighboring country of Vietnam [38]. Imipenem, meropenem, amikacin, and third-generation cephalosporins are still effective for most bacterial pathogens in the area. However, the use of imipenem and meropenem is not recommended in younger children. As a result, the treatment of choice in cases in which antibiotic therapy is recommended becomes a problem.

## Conclusions

Our results showed that in addition to rotavirus, calicivirus and DEC are important causative agents of childhood diarrhea in Zhejiang province. The high prevalence of rotavirus provides a scientific basis for rotavirus vaccines and other measures to prevent rotavirus infections. This knowledge of the broad spectrum of etiological agents of diarrhea in the participants will help us plan studies on the various aspects of diarrheal diseases in the Chinese population. However, the pathogen spectrum is dynamic, which emphasizes the need to carry out long-term monitoring. Much work remains to be done to decrease the prevalence of diarrhea in children less than 5 years of age in China.

## Abbreviations

CLSI, Clinical Laboratory Standards Institute; DEC, Diarrheagenic *E.coli*; EAEC, enteroaggregative *E. coli*; EIEC, enteroinvasive *E. coli*; EPEC, enteropathogenic *E. coli*; ESBL, extended-spectrum beta-lactamase; ETEC, enterotoxigenic *E. coli*; HucV, human calicivirus; PRC, The People’s Republic of China; STEC, Shiga toxin-producing *E. coli*
